# The influence of personality and perceived stress on the development of breast cancer: 20-year follow-up of 29,098 Japanese women

**DOI:** 10.1038/srep32559

**Published:** 2016-09-02

**Authors:** Takayuki Sawada, Takeshi Nishiyama, Norimasa Kikuchi, Chaochen Wang, Yingsong Lin, Mitsuru Mori, Kozo Tanno, Akiko Tamakoshi, Shogo Kikuchi

**Affiliations:** 1 Clinical Study Support, Inc, Nagoya, 460-0003, Japan; 2 Department of Public Health, Aichi Medical University School of Medicine, Nagakute, 480-1195, Japan; 3 Department of Public Health, Sapporo Medical University School of Medicine, Sapporo, 060-8556, Japan; 4 Department of Hygiene and Preventive Medicine, Iwate Medical University School of Medicine, Morioka, 028-3694, Japan; 5 Department of Public Health, Hokkaido University Graduate School of Medicine, Sapporo, 060-8638, Japan

## Abstract

Breast cancer is the most common cancer in women. However, it remains unproven whether psychological factors have an influence on breast cancer incidence. In our earlier study, subjects possessing two personality traits, decisiveness and “ikigai” (a Japanese word meaning something that makes one’s life worth living), showed a significantly lower risk of developing breast cancer, although no psychological factors have been convincingly demonstrated to have an influence on breast cancer development in other studies. Therefore, we conducted this follow-up analysis to confirm the association between breast cancer incidence and psychological traits, using the final dataset of a large-scale prospective cohort study in Japan. We identified 209 cases of incident breast cancer out of a maximum 21-year follow-up of 29,098 Japanese women. Cox proportional hazard regression analysis, adjusted for the same potential confounders used in our previous study, did not reveal any significant relationships between breast cancer incidence and four psychological traits: having “ikigai”, decisiveness, ease of anger arousal, and perceived stress. Our finding is consistent with previous studies, and suggests that the psychological traits are unlikely to be an important risk factor for breast cancer.

Breast cancer is the most common cancer in women worldwide^1^, as well as in Japan^2^. A number of breast cancer risk factors have been established, including age at menarche and menopause, obesity, height, regular exercise, nutrition, and family history of breast cancer^3^,^4^,^5^,^6^,^7^. In contrast, it remains unproven whether psychological factors have an influence on breast cancer incidence, though many cohort studies and systematic reviews have examined various psychological factors on this topic, including life events^8^,^9^,^10^, personality traits^11^,^12^,^13^,^14^,^15^,^16^,^17^, coping style^14^,^15^,^18^, perceived stress^19^,^20^, and depression^21^,^22^,^23^, over the past 30 years.

Two potential mechanisms are proposed to explain how these psychological factors work to increase cancer risk. The first is via altering the immune and endocrine systems, and the second is via altering behaviors such as sleep, excecise, diet, smoking, alchohol consumption, drug use, and poor adherence to medical regimens^24^,^25^. Studies evaluating relevance of psychological factors to carcinogenesis have most often examined the role of personality and stress as risk factors in cancer. Of the cancer sites that have been studied from this perspective, breast cancer is one that has received a great deal of attention^26^. Therefore, we focused on a possible contribution of personality traits and perceived stress to breast cancer development in the present study. In our earlier study, two psychological traits, “having something to live for” and “perceiving oneself as quick to judge”, showed a significantly lower risk of developing breast cancer. The other two traits, “ease of anger arousal” and “perceived stress”, did not show an association with breast cancer incidence^12^. Precisely, to indicate “something to live for”, we used the Japanese word, “ikigai” in the survey. Although “ikigai” has not been well defined in the scientific literature, this term is used to refers to 1) a specific experience that creates a sense of worth and happiness, 2) the resultant cognitive evaluation that finds one’s life meaning because of the experience, and 3) the sense of fulfillment and joy that is derived from the cognitive evaluation^27^. Thus, we used “ikigai” to represent this construct throughout the manuscript. Notably, this construct, “ikigai” was shown to be associated with mortality in another study using the same cohort in the present study^28^.

Recently, an elaborate meta-analysis using pooled data from six cohort studies revealed that personality was not associated with an increased risk of all cancers and site-specific cancers including breast cancer^11^. This meta-analysis was conducted on this topic comprehensively, using a general personality framework called the five-factor model, which is accepted in modern personality psychology^29^. As for perceived stress, the meta-analysis showed that stress-related psychosocial factors were not associated with an increased risk of breast cancer incidence^19^.

One limitation of these studies was the relatively short follow-up period (*e.g.*, mean follow-up of 5.4 years in the former meta-analysis and follow-up of 7–9 years in our previous study). Empirical studies demonstrated that it may take approximately 14 years for breast cancer to grow from the first tumor cell into a detectable lump and, thus, studies with these time frames are likely to underestimate associations with breast cancer incidence^30^,^31^. In fact, a meta-analysis found that cohort studies with a longer follow-up showed a stronger association between depression and breast cancer incidence^23^. Therefore, we conducted a maximum of 21 year follow-up analysis to confirm the association between breast cancer incidence and psychological traits using the final dataset of a large-scale prospective cohort study, the Japan Collaborative Cohort (JACC) Study^32^.

## Methods

### Study population

A baseline survey of the JACC Study was conducted in 45 areas throughout Japan. A total of 110,585 subjects (46,395 men and 64,190 women) aged 40–79 were enrolled between 1988 and 1990. The details of the study procedure were described elsewhere^32^.

This study excluded 23 study areas where information on cancer incidence at follow-up or psychological traits at baseline were not available. Furthermore, 247 subjects were excluded because of positive histories of breast cancer. Another 9,268 subjects were excluded because all of their data on psychological traits at baseline were missing. Thus, a total of 29,098 women were included in this study.

Informed consent was obtained individually from each participant, except in a few study areas where informed consent was obtained at the group level after the purpose of the study and confidentiality of the data had been explained to community leaders. The JACC Study begun before the ethical guideline was first established by Japanese government in 2002, and the Japanese ethical guideline allows such established epidemiological studies to continue without obtaining additional personal informed consents^33^,^34^,^35^. This study was approved by the ethics board at Nagoya University School of Medicine, where the central office of JACC Study was located^28^.

### Baseline Measuement

At baseline, we used a self-administered questionnaire to obtain information on demographics. medical history, tabacco and alcohol use, psychical activity, psychological traits, and other lifestyle factors. Psychological traits were measured using four questions at balseline: having “ikigai” (do you have “ikigai”, something to live for?), decisiveness (do you make decisions quickly?), ease of anger (are you easily angered?), and perceived stress (do you experience stress during your daily life?), with three- or four-point Likert-type response options (Table 1).

Potential confounders were also measured using the questionnaire at baseline; they are detailed in the statistical analysis section below.

### Outcome ascertainment

Follow-up was conducted from enrollment until the end of 2009 in all areas, except three areas where follow-up was terminated at the end of 1999. During this period, population registries in the municipalities were used to ascertain the residential and vital status of the subjects. In Japan, registration of death is required by Family Registration Law, and theoretically provides complete mortality data. The cause and date of death of subjects were annually or biannually confirmed by death certificates in each area. The date of move-out of cohort members from the study area was also annually or biannually verified by the investigator in cooperation with the local governmental office.

We ascertained the incidence of cancer by linking to records of population-based cancer registries, supplemented by a systematic review of death certificates. In some study areas, medical records in major local hospitals were also reviewed^36^.The cancer incidence data were coded according to the 10th Revision of the International Statistical Classification of Diseases and Related Health Problems (ICD-10). We defined breast cancer as C50.0 to C50.9, and do not classify breast cancer based on histopathology or molecular subtypes^37^.

### Statistical analysis

According to psychological traits responses, the baseline subject characteristics were summarized as percentage for categorical data and mean for continuous data with corresponding 95% confidence intervals (95% CI). Psychological trait variables were treated as ordinal variables for all analyses. and Spearman’s correlation coefficients among psychological traits were estimated with 95% CI. The person-years of follow-up were calculated from the baseline to the following events: diagnosis of breast cancer, death from any causes, emigration outside the study area, or the end of follow-up (the latter three events were treated as censored).

The hazard rations (HR) with the 95% CI for breast cancer incidence were estimated using Cox proportional hazards regression models. For estimating HR, we used age (using 10-year age groups) as an independent variable. We also used the following potential confounding variables used in the previous study^12^: age (using 10-year age groups); area (Hokkaido, Tohoku, Kanto, Chubu, Kinki, Chugoku, or Kyushu); educational level (attended school until the age of ≤15, 16–18, ≥19 years); family history of breast cancer in mother or sisters (yes or no); age at menarche (≤13, 14–15, 16–17 or ≥18 years); age at menopause (premenopausal at baseline, ≤44, 45–49 or ≥50 years); age at time of first birth (≤24, 25–29 or ≥30 years); number of times of giving birth or parity (0, 1, 2, 3 or ≥4); use of exogenous female hormones (yes or no); alcohol consumption (never drink, ex-drinker, or current drinker consuming <15 or ≥15 g of ethanol daily); consumption of green leafy vegetables (≤2 times/week, 3–4 times/week or almost every day); daily walking habits (seldom or never, or <30, 30–59 or ≥60 min/day); exercise (seldom or never, 1–2, 3–4 or ≥5 h/week); sedentary work (yes or no); height (<150.0, 150.0–159.9 or ≥160.0 cm); and baseline body-mass index (BMI; <20.0, 20.0–24.9, 25.0–29.9 or ≥30.0 kg/m^2^). In the Cox regression analysis, missing values for each covariate were treated as an additional category in the variable. The proportional hazards assumption was checked by visual inspection of the log-log Kaplan–Meier curves.

All *p*-values were two-sided, and the significance level was set to 0.05. All analyses were performed using SAS version 9.3 (SAS institute Inc., Cary, NC, USA).

## Results

Correlations are moderate among the four psychological traits as shown in Table 2, where the absolute values of Spearman’s correlation coefficients range from 0.03 to 0.32. Therefore, we examined each psychological trait separately for association with breast cancer incidence.

Baseline characteristics of the study population are summarized in Table 1 according to each psychological trait response. For each response regarding psychological traits, no change was shown in some of the characteristics, such as age at menarche, menopause and first birth, as well as parity and height, evidenced by overlapping 95% CI of these variables for each response. The other characteristics showed a contrast between having “ikigai” or decisiveness, and ease of anger or perceived stress. This general pattern was observed in the following variables: daily consumer of green leafly vegetables, walking, and exercise. Furthermore, use of exogenous female hormone was nearly aligned with this pattern of contrast, although exceptions occurred in the other variables. Of these exceptional variables, age, education, sedentary work, and BMI were almost identically distributed among all psychological traits.

During a maximum 21-year follow-up (a mean follow-up of 12.8 years, and 372,156 person-years), 209 cases of incident breast cancer were ascertained. As shown in Table 3, in the Cox regression analysis adjusted for sex, none of the psychological traits were significantly related to the risk of breast cancer. This finding remained basically unchanged after adjustment for the other potential confounders used in our previous study^12^. In the previous study, psychological traits responses were dichotomized for Cox regression analyses. Just to compare with the results from that study, therefore, we dichotomized the psychological traits responses for Cox regression. Again, none of the psychological traits were significantly related to the risk of breast cancer (Supplementary Table 1).

## Discussion

Our large-scale prospective cohort study with 19–21 years of follow-up did not reveal any significant relationships between breast cancer incidence and the four psychological traits: having “ikigai”, decisiveness, ease of anger arousal, and perceived stress.

In the previous study using the first 7–9 years follow-up data of the same cohort, two psychological traits. having “ikigai” and decisiveness, had a nominally significant association with breast cancer incidence^12^. However, this association disappeared after another 12 years of follow-up, even though three or four-point Likert-type responses were dichotomized in the same way as in the previous study (Supplementary Table 1). This inconsistency might have occurred because the previous study conducted statistical tests for four items, which led to type I error inflation. That is, the effects of psychological traits on the development of breast cancer are so negligible that the present study cannot detect them, but the previous study might have spuriously detected the effect due to multiple testing. Alternately, the inconsistent results might have occurred owing to different follow-up duration between our earlier and present studies. Because personality traits and perceived stress continue to change over time^38^,^39^,^40^, the longer follow-up duration, the weaker the effect of these traits on breast cancer incidence. Therefore, the present study with a longer follow-up might miss the effect of psychological traits that could be detected in the earlier study.

A clinically detectable tumor contains at least 10^9^ cells, and the doubling time for primary breast cancer tumors is estimated to be 105–270 days^30^,^41^; thus, it should take 8.6–13.9 years to grow from the first tumor cell to a detectable tumor. Therefore, to allow for a sufficient incubation period of breast cancer, we focused on previous cohort studies with more than 13 years of follow-up that examined the association between breast cancer incidence and any personality traits. To our knowledge, there are three such studies, and all of them revealed no significant increase of breast cancer risk by any personality traits examined^13^,^16^,^17^. Considering these findings together with the results of the present study, the overall findings are largely reassuring that the psychological traits do not have a substantial role in the etiology of breast cancer.

The construct “ikigai” indicates the source of value in one’s life or the things that make one’s life worthwhile. This construct is noteworthy because it was repeatedly shown to be associated with mortality in another study using the same cohort in the current^28^ and other studies^42^,^43^,^44^. In these studies, the increase in mortality was attributed to an increase in the mortality from cardiovascular disease and external causes, but not to the mortality from cancer^28^,^42^,^43^. The latter finding is in agreement with the present study. This study had some limitations. The most important limitation concerns the measures of the four constructs examined here. We used only one item to measure each construct. Compared to a multiple-item measure, single-item measures might be more influenced by random measurement error, which could attenuate the true association between breast cancer incidence and each item and, thus, decrease the power to detecting the association^45^. This may be one of the reasons why we failed to obtain any significant results. Furthermore, these single-item measures were not shown to be valid, although they have face validity. That is, we are not confident in what these items measure in fact. Especially, the definition of “ikigai” has not been agreed upon in the scientific literature, and its construct validity still needs to be established. Another measurement problem is that the four psychological traits were measured only at baseline. This approach cannot address the influence of the traits over time, and thus a repeated measures design could uncover its influence on breast cancer incidence that would otherwise be missed. Finally, for identification of breast cancer in the present study, population-based cancer registries were not available in four out of 24 study areas; instead, medical records in local hospitals were reviewed in the four areas. The unsystematic identification of cancer cases in the small portion of study areas could cause a systematic error.

Therefore, future studies should follow-up for a period long enough to grasp the whole picture of breast cancer development, using well-validated questionnaires to measure psychological traits.

## Conclusion

This large-scale prospective cohort study failed to find any significant association between the four psychological traits examined and breast cancer incidence in Japan. The fact that the psychological traits are unlikely to be an important risk factor for breast cancer may be a comfort to women who are otherwise concerned about the risk of personality traits leading to breast cancer.

## Additional Information

**How to cite this article**: Sawada, T. *et al*. The influence of personality and perceived stress on the development of breast cancer: 20-year follow-up of 29,098 Japanese women. *Sci. Rep.* **6**, 32559; doi: 10.1038/srep32559 (2016).

## Supplementary Material

Supplementary Information

## Figures and Tables

**Table 1:** Baseline characteristics of subjects according to psychological item responses.

Item	Having ‘ikigai'				Decisiveness			Ease of anger			Perceived stress			
Response	Agree strongly	Agree	Neither	Disagree	Agree	Neither	Disagree	Agree	Neither	Disagree	Agree strongly	Agree	Neither	Disagree
Age^a^	57.2(56.9–57.6)	56.7(56.5–56.9)	57.9(57.8–58.1)	58.5(58.0–59.0)	56.1(55.9–56.3)	57.6(57.5–57.8)	59.5(59.1–59.9)	55.3(55.0–55.7)	57.4(57.3–57.6)	59.9(59.5–60.2)	54.2(53.8–54.5)	54.8(54.5–55.2)	58.1(57.9–58.2)	59.3(59.0–59.6)
Education > high school^b^	16.9%(15.5–18.4)	13.4%(12.7–14.1)	9.3%(8.8–9.8)	8.4%(7.2–9.7)	15.3%(14.5–16.3)	10.4%(9.9–10.8)	8.8%(7.8–9.9)	12.5%(11.4–13.6)	11.1%(10.7–11.5)	11.5%(10.5–12.6)	13.3%(12.0–14.6)	15.6%(14.3–16.9)	10.2%(9.7–19.6)	11.7%(10.8–12.7)
Family history^c^	2.0%(1.5–2.6)	1.7%(1.4–2.0)	1.5%(1.3–1.8)	1.0%(0.6–1.6)	1.8%(1.5–2.1)	1.6%(1.4–1.8)	1.3%(0.9–1.8)	1.7%(1.4–2.2)	1.6%(1.4–1.8)	1.5%(1.1–1.9)	1.7%(1.3–2.2)	2.2%(1.7–2.7)	1.4%(1.3–1.6)	1.8%(1.4–2.2)
Age at menarche^a^	14.8(14.7–14.9)	14.7(14.7–14.8)	14.8(14.8–14.8)	15.0(14.9–15.1)	14.7(14.7–14.8)	14.8(14.8–14.8)	14.9(14.9–15.0)	14.7(14.6–14.8)	14.8(14.7–14.8)	14.9(14.9–15.0)	14.6(14.5–14.7)	14.5(14.4–14.6)	14.9(14.8–14.9)	14.8(14.8–14.9)
Menopause	69.9%(68.2–71.6)	67.7%(66.7–68.6)	69.8%(69.0–70.5)	64.0%(62.0–66.1)	65.5%(64.4–66.6)	69.3%(68.6–69.9)	72.0%(70.4–73.5)	62.4%(60.9–64.0)	68.9%(68.3–69.5)	73.5%(72.1–74.8)	59.5%(57.7–61.3)	62.0%(60.3–63.7)	70.1%(69.5–70.8)	73.4%(72.1–74.6)
Age at menopause^a^	48.7(48.5–49.0)	48.8(48.7–48.9)	48.7(48.6–48.8)	48.2(48.0–48.5)	48.6(48.4–48.7)	48.8(48.7–48.8)	48.6(48.4–48.8)	48.4(48.2–48.6)	48.7(48.7–48.8)	48.8(48.6–49.0)	48.4(48.1–48.6)	48.5(48.2–48.7)	48.7(48.6–48.8)	48.9(48.7–49.0)
Age at first birth^a^	25.0(24.9–25.1)	25.0(24.9–25.0)	25.1(25.0–25.2)	25.1(24.9–25.2)	25.0(24.9–25.0)	25.0(25.0–25.1)	25.2(25.1–25.3)	25.1(25.0–25.2)	25.0(25.0–25.1)	25.1(25.0–25.2)	25.2(25.1–25.4)	25.1(24.9–25.2)	25.0(25.0–25.1)	25.0(24.9–25.1)
Partity	2.6(2.6–2.7)	2.6(2.6–2.6)	2.6(2.6–2.6)	2.7(2.6–2.7)	2.5(2.5–2.5)	2.6(2.6–2.6)	2.8(2.7–2.8)	2.5(2.4–2.5)	2.6(2.6–2.6)	2.7(2.7–2.7)	2.4(2.4–2.5)	2.5(2.5–2.5)	2.6(2.6–2.7)	2.6(2.6–2.7)
female hormone use^d^	7.2%(6.3–8.3)	7.2%(6.7–7.7)	9.5%(9.0–10.0)	10.4%(9.1–11.7)	7.3%(6.7–8.0)	8.8%(8.4–9.2)	9.8%(8.8–10.9)	8.1%(7.3–9.0)	8.6%(8.3–9.0)	8.6%(7.8–9.5)	9.4%(8.3–10.5)	6.8%(6.0–7.7)	9.1%(8.7–9.5)	7.1%(6.4–7.9)
Current drinkers	32.4%(30.6–34.2)	27.6%(26.7–28.6)	23.0%(22.3–23.7)	20.3%(18.6–22.2)	31.1%(30.0–32.2)	24.0%(23.4–24.6)	20.4%(19.0–21.9)	30.5%(29.0–32.0)	24.7%(24.1–25.3)	23.3%(22.0–24.7)	27.8%(26.1–29.5)	28.7%(27.1–30.3)	23.9%(23.3–24.6)	26.6%(25.3–27.9)
Former drinkers	2.0%(1.5–2.6)	1.7%(1.5–2.0)	1.7%(1.5–1.9)	2.8%(2.1–3.6)	2.2%(1.8–2.6)	1.7%(1.5–1.9)	1.8%(1.4–2.4)	2.8%(2.3–3.3)	1.6%(1.4–1.8)	2.1%(1.7–2.6)	2.6%(2.0–3.2)	2.2%(1.7–2.8)	1.6%(1.4–1.8)	2.0%(1.6–2.5)
Daily intake of green leaf	31.7%(29.9–33.4)	34.6%(33.7–35.6)	41.3%(40.5–42.1)	47.5%(45.4–49.7)	35.2%(34.1–36.4)	38.6%(37.9–39.3)	45.2%(43.5–47.0)	42.1%(40.6–43.7)	38.4%(37.7–39.1)	36.0%(34.6–37.5)	42.4%(40.6–44.2)	38.2%(36.5–39.9)	39.1%(38.4–40.0)	34.5%(33.1–35.9)
Walking > 30 min/day	76.5%(74.8–78.1)	73.8%(72.9–74.7)	70.5%(69.7–71.2)	63.5%(61.3–65.6)	72.4%(71.4–73.6)	72.4% (71.8–73.1)	65.6%(63.9–67.3)	67.7%(66.2–69.2)	72.3%(71.7–73.0)	72.1%(70.7–73.5)	70.2%(68.4–71.8)	70.2%(68.6–71.8)	72.1%(71.4–72.8)	72.1%(70.7–73.4)
Exercise ≥ 3 h/week	16.1%(14.7–17.5)	11.3%(10.7–12.0)	7.9%(7.4–8.4)	6.0%(5.0–7.1)	11.7%(11.0–12.5)	9.4%(9.0–9.8)	7.0%(6.1–8.0)	9.0%(8.0–9.8%)	9.6%(9.2–10.0)	11.1%(10.1–12.1)	7.3%(6.4–8.4)	8.4%(7.5–9.5)	9.8%(9.4–10.3)	11.5%(10.6–12.5)
Sedentary work	35.2%(33.3–37.2)	35.8%(34.8–36.9)	35.0%(34.2–35.9)	32.8%(30.5–35.1)	38.5%(37.3–39.8)	34.3%(33.5–35.0)	33.0%(31.2–34.9)	37.5%(35.8–39.2)	34.7%(34.0–35.4)	35.1%(33.4–36.7)	40.7%(38.7–42.6)	37.6%(35.8–39.4)	33.7%(32.9–34.5)	35.3%(33.8–36.9)
Height (cm)^e^	152.0(151.7–2.3)	151.9(151.8–2.0)	151.2(151.1–1.3)	150.7(150.4–0.9)	152.4(152.3–2.6)	151.3(151.2–1.4)	150.2(149.9–0.4)	152.1(151.9–2.3)	151.4(151.4–1.5)	151.0(150.8–1.2)	152.2(152.0–2.4)	152.3(152.1–2.5)	151.3(151.2–1.4)	151.2(151.0–1.4)
Body mass index(kg/m^2^)	23.1(22.9–23.3)	22.8(22.7–22.9)	22.7(22.7–22.8)	22.6(22.4–22.7)	23.0(22.9–23.1)	22.8(22.7–22.8)	22.5(22.4–22.6)	22.9(22.8–23.0)	22.8(22.7–22.8)	22.8(22.7–22.9)	22.8(22.7–22.9)	22.6(22.5–22.7)	22.8(22.8–22.9)	22.8(22.7–22.9)

95% CI is reported in parentheses.

^a^Age is measured in year.

^b^Education beyond high school.

^c^Family history of breast cancer in mother and/or sisters.

^d^Ever used exogenous female hormone.

^e^Upper limit of 95% CI is shown with a value after subtracting 150 (cm).

**Table 2:** Spearman’s correlation coefficients among the four items for psychological characteristics.

Psychological traits	Questions	“Ikigai”	Decisiveness	Ease of anger arousal	Psychological stress
“Ikigai”	(Do you have “ikigai”?)	—	0.29 (0.28, 0.30)	−0.08 (−0.09, −0.07)	−0.15 (−0.16, −0.13)
Decisiveness	(Do you make decisions quickly?)	—	—	0.03 (0.02, 0.04)	−0.04 (−0.05, −0.02)
Ease of anger arousal	(Are you easily angered?)	—	—	—	0.33 (0.32, 0.34)
Psychological stress of daily life	(Do you experience stress during your daily life?)	—	—	—	—

95% CI was estimated using Fisher’s Z transformation, and is shown in parentheses.

**Table 3:** Hazard ratios(HR) with 95% confidence intervals(CI) for breast cancer incidence according to psychological item responses.

Response to questions	No. of women	Observed person-years	No. of cases	Age-adjusted		Multivariate-adjusted^a^	
				HR	95%CI	HR	95%CI
Having “ikigai”							
Disagree	2,068	25,355	15	1.00		1.00	
Neither	14,505	185,952	114	1.03	0.61–1.77	1.02	0.59–1.75
Agree	9,770	125,897	61	0.80	0.46–1.41	0.77	0.43–1.37
Agree strongly	2,755	34,952	19	0.90	0.46–1.78	0.81	0.41–1.62
Decisiveness							
Disagree	3155	38,349	18	1.00		1.00	
Neither	19,206	247,330	140	1.19	0.73–1.94	1.18	0.72–1.94
Agree	6,737	86,477	51	1.21	0.71–2.08	1.07	0.62–1.85
Ease of anger							
Disagree	4,171	51,898	22	1.00		1.00	
Neither	21,013	270,672	163	1.38	0.88–2.16	1.41	0.91–2.21
Agree	3,914	49,585	24	1.08	0.61–1.94	0.98	0.55–1.76
Perceived stress							
Disagree	4,730	57,634	28	1.00		1.00	
Neither	18,208	235,880	128	1.11	0.74–1.67	1.21	0.80–1.82
Agree	3,214	38,859	33	1.66	1.00–2.76	1.71	1.02–2.85
Agree strongly	2,946	39,782	20	1.00	0.56–1.78	1.00	0.56–1.78

^a^Adjusted for age, study area, educational level, family history of breast cancer, age at menarche, age at menopause, age at first birth, parity, use of exogenous female hormone, alcohol drinking, consumption of green leafy vegetables, daily walking, exercise, sedentary work, height, and body mass index
