# Supplementary material for: The influence of personality and perceived stress on the development of breast cancer: 20-year follow-up of 29,098 Japanese women
Source: Sci Rep. 2016 Sep 2;6:32559. doi: 10.1038/srep32559 (PMC5009304; doi:10.1038/srep32559)
Supplement: Supplementary Information [file srep32559-s1.pdf]

Scientific Reports

The influence of personality and perceived stress on the development of breast cancer: 20-year follow-up of 29,098 Japanese women

Takayuki Sawada[1,2], Takeshi Nishiyama[2,*], Norimasa Kikuchi[1,2], Chaochen Wang[2], Yingsong Lin[2], Mitsuru Mori[3], Kozo Tanno[4], Akiko Tamakoshi[5], Shogo Kikuchi[2,*]

1) Clinical Study Support, Inc, Nagoya, 460-0003, Japan

2) Department of Public Health, Aichi Medical University School of Medicine, Nagakute, 480-1195, Japan

3) Department of Public Health, Sapporo Medical University School of Medicine, Sapporo, 060-8556, Japan

4) Department of Hygien and Preventive Medicine, Iwate Medical University School of Medicine, Morioka, 028-3694, Japan

5) Department of Public Health, Hokkaido University Graduate School of Medicine, Sapporo, 060-8638, Japan

Supplementary Table 1. Hazard ratios(HR) with 95% confidence intervals(CI) for breast cancer by response to psychological questions

Response to questions	No. of women	Observed person-years	No. of cases	Age-adjusted		Multivariate-adjusted[a]	
				HR	95% CI	HR	95% CI
Having "ikigai"							
Do you have "ikigai"?							
Disagree/Neither	16,573	211,307	129	1.00		1.00	
Agree/Agree strongly	12,525	160,849	80	0.80	0.61-1.06	0.77	0.58-1.02
Decisiveness							
Do you make decisions quickly?							
Disagree/Neither	22,361	285,679	158	1.00		1.00	
Agree	6,737	86,477	51	1.04	0.76-1.43	0.92	0.67-1.28
Ease of anger arousal							
Are you easily angered?							
Disagree/Neither	25,184	322,571	185	1.00		1.00	
Agree	3,914	49,585	24	0.82	0.53-1.25	0.73	0.48-1.12
Psychological stress							
Do you experience stress during your daily life?							
Disagree/Neither	22,938	293,515	156	1.00		1.00	
Agree/Agree strongly	6,160	78,641	53	1.22	0.89-1.68	1.16	0.84-1.59

[a]Adjusted for age, study area, educational level, family history of breast cancer, age at menarche, age at menopause, age at first birth, parity, use of exogenous female hormone, alcohol drinking, consumption of green leafy vegetables, daily walking, exercise, sedentary work, height, and body mass index
